# Anomalous Origin of the Right Coronary Artery From the Pulmonary Artery: Advantages of Multimodality Imaging

**DOI:** 10.7759/cureus.64294

**Published:** 2024-07-10

**Authors:** Andreia D Constante, Petra Loureiro, José F Martins, Fátima Pinto

**Affiliations:** 1 Pediatric Cardiology Department, Unidade Local de Saúde São José, Hospital de Santa Marta, Lisbon, PRT

**Keywords:** coronary re-implantation, anomalous origin of the right coronary artery from the pulmonary artery, multimodality imaging, anomalous coronary arteries, arcapa

## Abstract

Coronary anomalies are one of the most surprising yet challenging pediatric cardiology diagnoses. The anomalous origin of the right coronary artery from the pulmonary artery (ARCAPA) is frequently underdiagnosed due to a lack of typical signs or symptoms. We present a case of ARCAPA in a healthy six-month-old girl during follow-up of a newly detected heart murmur. Echocardiography raised the suspicion of a coronary anomaly, but the diagnosis was unclear, so cardiac catheterization and computed tomography were performed, which posteriorly confirmed the diagnosis. The patient underwent surgical repair, and the short-term follow-up has been uneventful. Regular monitoring is essential due to the potential long-term complications of ARCAPA, including myocardial ischemia, heart failure, and sudden cardiac death, underscoring the importance of early diagnosis and continuous management.

## Introduction

Coronary anomalies are one of the most surprising yet challenging diagnoses in pediatric cardiology. Within this group, the anomalous origin of the right coronary artery (RCA) from the pulmonary artery (ARCAPA) is an exceptionally rare congenital coronary anomaly, representing about 0.12% of all coronary anomalies with an estimated incidence in the literature of 0.002% [1]. We report a case of a six-month-old girl with an incidental diagnosis of ARCAPA, describing the entire diagnostic workup from clinical suspicion to surgical intervention.

## Case presentation

**Figure 1:**
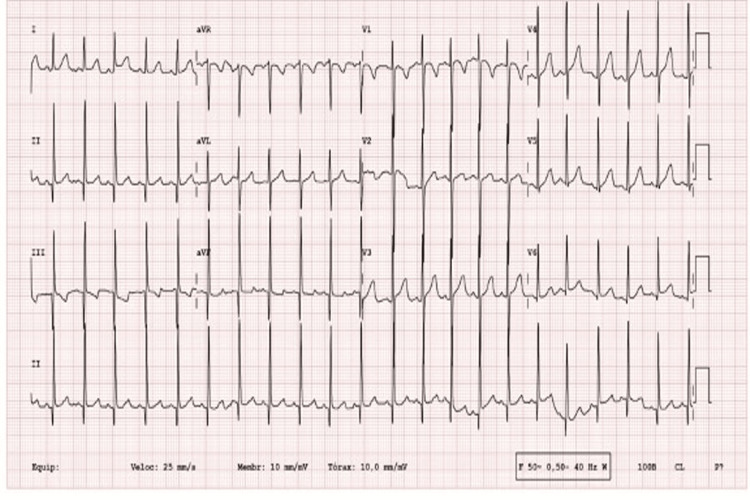
ECG showing deep Q waves in the inferior leads.

**Figure 2:**
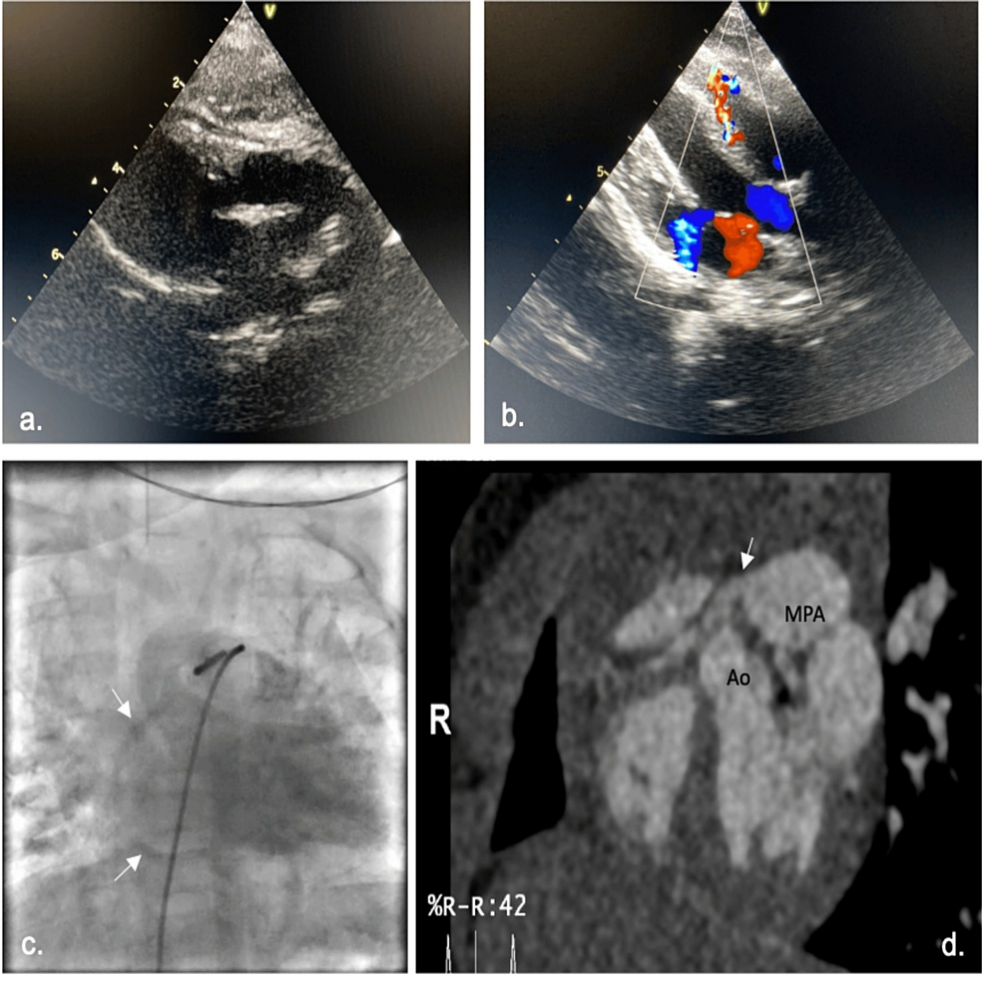
Multimodality imaging workup for the diagnosis of anomalous right coronary artery from the pulmonary artery. (a and b) Echocardiogram demonstrating the course of the RCA above the aorta and color flow Doppler revealing an anomalous coronary branch into the right ventricle (due to collateral circulation) with mitral regurgitation. (c) MPA angiography injection revealing ARCAPA (white arrows). (d) Angio-CT shows RCA (white arrow) arising from the MPA. Ao: aorta; ARCAPA: anomalous origin of the right coronary artery from the pulmonary artery; MPA: main pulmonary artery; RCA: right coronary artery.

A previously healthy six-month-old infant was referred to our Pediatric Cardiology consultation for a newly recognized heart murmur. She exhibited good height-weight evolution and had no complaints. On physical exam, she was well nourished, eupneic, and had a continuous murmur along the left sternal border with no particular irradiation pattern, good femoral and peripheral pulses, and no hepatomegaly. The ECG showed deep Q waves in DIII and augmented vector foot (aVF) (Figure 1). The initial echocardiogram suggested a coronary fistula from the anterior descending artery to the right ventricle (RV), mild mitral regurgitation but normal-sized atria and ventricles, and no evidence of additional structural anomalies (Figure 2a,b). Echocardiographic re-evaluation revealed retrograde blood flow in the RCA. She underwent diagnostic cardiac catheterization for better characterization of the coronary defect. Contrast injection into the root of the aorta showed the left coronary artery (LCA) with normal origin and distribution and the RCA with late filling; the ostium was not located in the aorta. Injections in the pulmonary artery showed ARCAPA (Figure 2c). No coronary fistulas were observed. Computed tomography angiography confirmed that the RCA originated from the pulmonary artery trunk and coursed towards the right coronary sinus (Figure 2d). The patient underwent right coronary re-implantation surgery. The postoperative period was uneventful, without evidence of myocardial ischemia. She was discharged on the fourth postoperative day in good clinical condition and is under a structured follow-up plan involving periodic cardiology visits and echocardiography studies to monitor the long-term success of the surgical repair and ensure early detection of any potential complications.

## Discussion

ARCAPA, although benign in its presentation (mostly asymptomatic), has an underlying deleterious potential in the development of chest pain, arrhythmias, syncope, acute myocardial infarction, and, ultimately, sudden death [2]. A recent systematic review showed a total of 223 cases of ARCAPA described in the literature. A relevant percentage of the patients (38%) were asymptomatic and most commonly identified during the assessment of a murmur. In almost a quarter of the cases, it appears to be associated concomitantly with another cardiac lesion, with the aortopulmonary window being the most common of all [3].

Patients with ARCAPA tend to be diagnosed later than those with anomalous origin of the left coronary artery from the pulmonary artery (ALCAPA), as the latter are often more symptomatic and detected earlier in life [4]. The initial diagnostic approach begins with transthoracic echocardiography (TTE), which visualizes retrograde flow in the RCA predominantly in diastole, as observed in our case. However, other signs include collateralization, the presence of a dilated RCA, increased flow in the LCA, and visualization of flow between the RCA and the pulmonary artery. The mild mitral regurgitation was another feature that initially raised the suspicion of a coronary anomaly. TTE, although useful as a first-line exam, is not always straightforward for assessing coronary anomalies or differentiating them.

Coronary angiography has been considered the “gold standard” for the evaluation of coronary anomalies due to its anatomical characterization capacity. In ARCAPA, it allows the identification of important features such as retrograde flow through the RCA, collateralization between the LCA and RCA, increased flow in the LCA/RCA, and eventually the presence of direct flow between the RCA and the pulmonary artery [3]. It has excellent spatial and temporal resolution, but its use is limited by technical and functional constraints in very young and critically ill children. CT-Angio has emerged as a capable and more reliable alternative for identifying these anomalies [5]. In 2014, a study by Ghadri et al. demonstrated that the prevalence of coronary anomalies is substantially higher with CT-Angio than with conventional angiography [6].

The option for surgical treatment in our patient was based on the information described in the literature, in which the majority opt for correction even in asymptomatic individuals, mitigating the long-term risks of ischemia and sudden death [7,8]. The treatment strategies that are used in patients with ARCAPA involve (1) ligation only or (2) re-establishment of the dual coronary artery system, which can be accomplished by aortic re-implantation, intrapulmonary tunnel shunt repair, or ligation with coronary artery bypass grafting (CABG). Aortic re-implantation is the most commonly performed surgical technique, even in asymptomatic patients who were incidentally diagnosed with ARCAPA, with most centers suggesting elective repair in an attempt to avoid future complications [3].

## Conclusions

This case highlights the utility of multimodality imaging techniques in the diagnosis of coronary anomalies for their accuracy and better anatomical characterization, allowing a timely surgical approach and improved clinical outcomes.
